# Efficacy and safety of telitacicept in children with IgA vasculitis and IgA vasculitis nephritis: a single-center retrospective study

**DOI:** 10.1186/s12969-025-01159-3

**Published:** 2025-10-21

**Authors:** Xueqing Ma, Yonghua He, Panpan Shao, Ling Guo, Wenpei Liang, Jianhua Zhou, Huiqing Yuan, Liru Qiu

**Affiliations:** Department of Pediatrics, Hubei Provincial Key Laboratory of Pediatric Genetic Metabolic and Endocrine Rare Diseases, Hubei Provincial Clinical Research Center for Children’s Growth and Development and Metabolic Diseases, Tongji Hospital, Tongji Medical College, Huazhong University of Science and Technology, Wuhan, 430030 China

**Keywords:** IgA vasculitis, IgA vasculitis nephritis, Talitacicept, Children

## Abstract

**Background:**

Immunoglobulin A vasculitis (IgAV) is the most common childhood vasculitis and can lead to immunoglobulin A vasculitis nephritis (IgAVN) in severe cases, potentially progressing to kidney failure in a subset of children. Safer and more effective treatments are needed to improve outcomes in these children. This study aimed to evaluate the efficacy and safety of telitacicept in the treatment of children with IgAV and IgAVN.

**Methods:**

This is a single-center, retrospective observational study of twenty four children with IgAV or IgAVN who received telitacicept treatment, and thirty matched children with IgAVN who only received conventional treatment were taken as the control group for children with IgAVN who received telitacicept treatment in acute phase. The treatment response was evaluated through urine protein, serum albumin, eGFR and serum immunoglobulin levels, and data was analyzed at telitacicept initiation and at 4, 12, 24 and 36 weeks after treatment.

**Results:**

A total of twenty four children (thirteen boys and eleven girls) with IgAV (*n* = 5) and IgAVN (*n* = 19, comprising ten acute and nine chronic cases) were enrolled. All children with IgAV experienced improvement of skin, joint, and gastrointestinal symptoms after telitacicept treatment, with no kidney involvement during follow-up. In children with IgAVN, the urinary protein-to-creatinine ratio (UPCR) significantly decreased at 36 weeks compared to baseline (*P* < 0.05) in both acute and chronic groups, while estimated glomerular filtration rate (eGFR) remained stable (*P* > 0.05). In addition, the dose of steroids administered during the treatment with telitacicept was significantly reduced, the acute IgAVN group exhibited significantly greater steroid reduction between weeks 4 and 24 compared with the controls group (*P* < 0.05). Furthermore, serum immunoglobulin levels (IgA, IgG) significantly decreased 12 weeks after telitacicept treatment (*P* < 0.01), and no other adverse reactions observed.

**Conclusion:**

Telitacicept appears to be a promising therapy for children with IgAV and IgAVN, effectively inducing proteinuria remission, improving systemic symptoms, and reducing the use of steroids, with favorable safety.

**Supplementary Information:**

The online version contains supplementary material available at 10.1186/s12969-025-01159-3.

## Introduction

Immunoglobulin A vasculitis (IgAV), formerly known as Henoch-Schönlein purpura, is the most common vasculitis in children [1]. IgAV is characterized by IgA-dominant immune deposits, often involving the skin, joints, gastrointestinal tract, and kidneys. When kidney involvement occurs, it is termed Immunoglobulin A vasculitis nephritis (IgAVN). Although IgAV is generally considered a self-limiting disease with a favorable prognosis, an increasing number of studies have shown that some children with IgAVN may progress to kidney failure, which is the main cause of long-term mortality in these children [2, 3]. Bogdanovic, R. et al. [4] conducted a long-term follow-up study on children with IgAVN and found that 1–2% of the children would develop into CKD over a long course of the disease. Goldstein, A.R. et al. [5] found that up to 20–30% of children progressed to chronic kidney disease(CKD) or End Stage of Kidney Disease (ESKD) 20 years after the diagnosis of IgAV. Children with IgAVN rarely developed renal failure in childhood. However, acute exacerbation after multiple infections can cause chronic kidney damage and long-term vascular lesions [6, 7]. Currently, there is no universally accepted standardized therapy for IgAVN. The treatment of children with IgAVN is mostly based on the clinical practice guidelines for the diagnosis and management of IgA nephropathy (IgAN) and IgAVN in children issued by IPNA [8] and the clinical practice guidelines for the management of IgAN and IgAVN in KDIGO (Draft for Public Review) [9]. They emphasize early detection, biopsy in severe/proteinuric cases, and tailored immunosuppression. Corticosteroids and immunosuppressants, such as cyclophosphamide(CTX), mycophenolate mofetil(MMF), calcineurin inhibitors(CNIs), or azathioprine are commonly used in moderate to severe cases. However, prolonged high-dose steroid use can lead to significant adverse effects in children (e.g., growth retardation, osteoporosis, metabolic disorders), and the efficacy of traditional immunosuppressive regimens is inconsistent. Therefore, safer and more effective drugs are being explored for children with IgAVN.

The pathogenesis of IgAVN is thought to be closely related to abnormal IgA immune complex formation. A key initiating factor is galactose-deficient IgA1 (Gd-IgA1), which can form immune complexes that deposit in glomeruli [10]. B cells play a key role in the production of Gd-IgA1 and the progression of IgAVN. B cell activating factor (BAFF, also known as BLyS) and a proliferation-inducing ligand (APRIL) are critical factors in the development and maturation of B cells, participating in the differentiation of B cells into plasma cells, antibody secretion, and immune complex formation [11, 12]. Blocking BAFF and APRIL may modulate the abnormal immune response in IgAVN. Telitacicept, a fusion protein targeting both BAFF and APRIL, has shown efficacy in treating autoimmune and rheumatic diseases. Clinical studies have demonstrated that telitacicept improves kidney function in patients with lupus nephritis and IgAN [13–15]. Given the mechanistic overlap between IgAN and IgAVN (both characterized by Gd-IgA1 immune complex and mesangial deposition), telitacicept is a promising candidate for treating IgAVN.

Recently, several case series and real-world studies have emerged indicating that telitacicept might be effective for refractory IgAVN in children. Liu et al. [16] reported that adding telitacicept reduced proteinuria by 50 ~ 80% in children with refractory IgAVN or IgAN. Jin et al. [17] and Wang et al. [18] likewise documented significant proteinuria reduction and clinical improvement with telitacicept in children with difficult-to-treat IgAVN. The vast majority of studies in the current literature focus on patients with refractory IgAVN, in whom telitacicept represents a valid therapeutic opportunity. Its use in children with acute IgAVN (within six months of onset) or without nephritis has not been widely studied, and the available evidence in these populations remains limited. Further well-designed studies are required to clarify its safety and efficacy in these settings. In this study, we present a retrospective analysis to evaluate the efficacy and safety of telitacicept in children with IgAV and IgAVN. We particularly assess its role in inducing remission of IgAVN (including both acute and chronic, refractory cases), its steroid-sparing effect, and any potential to prevent the progression of IgAV to nephritis.

## Methods

### Patients

A retrospective observational study was conducted in children with IgAV or IgAVN who received telitacicept treatment at the Department of Pediatric Nephrology, Tongji Hospital, Tongji Medical College, Huazhong University of Science and Technology between November 2023 and November 2024. Inclusion criteria:1) Age < 18 years; 2) Patients with IgAV fulfilling the Ankara 2008 criteria [19] and presenting with high-risk features, including persistent severe purpura, arthritis/arthralgia, or severe gastrointestinal manifestations unresponsive to glucocorticoids; 3) Biopsy-proven IgAVN patients who developed severe extrarenal manifestations (gastrointestinal or joint symptoms) within 6 months and were refractory to glucocorticoids or conventional immunosuppressants; 4) Biopsy-proven IgAVN patients with relapse despite ≥ 6 months of standard therapy, defined as recurrent gross hematuria, persistent proteinuria >0.5 g/1.73 m²/day, or progressive renal dysfunction (eGFR decline ≥ 5 ml/min/1.73 m²/year), refractory or intolerant to glucocorticoids and/or immunosuppressants (e.g., cyclophosphamide, mycophenolate mofetil, calcineurin inhibitors). Exclusion criteria: (1) Use of other B-cell targeted biologics (e.g., rituximab) within 3 months prior to telitacicept initiation. (2) Patients with a history of organ transplantation.

According to the meta-analysis by Chan H et al. [20], abdominal pain, gastrointestinal bleeding, severe bowel angina and persistent purpura are significant risk factors associated with renal involvement in IgAV. Arthritis/arthralgia may be a risk factor according to the criteria of the American College of Rheumatology [21]. Transient hematuria or proteinuria, has been associated with a higher likelihood of renal manifestations during the disease course [22].

Patients were categorized into several groups as follows:

IgAV group: (1) The patient is diagnosed with IgAV and has high-risk factors for kidney involvement, including severe purpura, abdominal pain, gastrointestinal bleeding, severe bowel angina, arthritis/arthralgia, and abnormal urinalysis; (2) The patient has no response to or is intolerant of glucocorticoids. IgAV group patient needs to meet both criteria (1) and (2).

Acute IgAVN group: (1) Definite diagnosis of IgAVN confirmed by renal biopsy, showing mesangial IgA deposition with glomerular injury; (2) The patient with sever extrarenal symptoms including severe purpura, abdominal pain, gastrointestinal bleeding, severe bowel angina, arthritis/arthralgia; (3) The patient has no response to or is intolerant of glucocorticoids and/or immunosuppressants (e.g., CTX, MMF, calcineurin inhibitors). The acute IgAVN group patient needs to meet criteria (1) (2) (3).

Chronic IgAVN group: (1) Definite diagnosis of IgAVN confirmed by renal biopsy, showing mesangial IgA deposition with glomerular injury; (2) Persistent proteinuria ≥ 0.5 g/m²/24 h in children despite ≥ 6 months of standard therapy or hematuria with proteinuria and progressive renal dysfunction (eGFR decline > 5 ml/min/1.73 m² per year); (3) Refractory or intolerant to glucocorticoids and/or immunosuppressants (e.g., CTX, MMF, calcineurin inhibitors). The chronic IgAVN group patient needs to meet criteria (1) (2) or (1) (3).

Control group: To assess outcomes compared to conventional treatment, we identified a comparison cohort for the acute IgAVN group. From the center’s medical record database, children diagnosed with IgAVN between January 2011 and December 2017 who had not received telitacicept were matched to the acute IgAVN group by propensity score matching (PSM). Propensity scores were estimated using logistic regression including baseline covariates (age, gender and kidney pathology). Patients were matched at a 1:3 ratio using nearest-neighbor matching without replacement, with a caliper of 0.2 of the standard deviation of the logit of the propensity score. The control group received conventional therapy according to the “Evidence-based guideline on diagnosis and treatment of Henoch-Schonlein purpura nephritis " [23] published in China in 2009 and KDIGO (2012) - Chap. 11: Therapeutic recommendations of Henoch-Schonlein Purpura Nephritis for HSPN in children [24].

### Study protocol

1. Telitacicept administration: Telitacicept was administered subcutaneously once weekly. The dosage was 80 mg for children weighing 20–40 kg and 160 mg for those weighing > 40 kg. Telitacicept was administered concurrently with conventional therapy. Physician determined the use of immunosuppressants and the reduction of steroids.

2. Data collection: Clinical data were collected through the medical record system of Tongji Hospital, Tongji Medical College, Huazhong University of Science and Technology, including gender, age, disease duration, clinical manifestations, laboratory examination, pathological findings, treatment, and follow-up data (monthly follow-up via phone or internet until March 2025). Urinary protein which quantified as 24-hour urinary protein (24-hUP) or urine protein-to-creatinine ratio (UPCR) (Spot UPCR samples were collected from the first morning void whenever possible, in order to minimize diurnal variation. In cases where this was not feasible, random spot urine samples were used), serum albumin, estimated glomerular filtration rate (eGFR) calculated by the Schwartz formula, and serum immunoglobulin levels (IgA, IgG, IgM) were recorded at baseline (start of telitacicept) and during follow-up at approximately 4 weeks, 12 weeks, 24 weeks, and 36 weeks of therapy. Define clinically significant hypogammaglobulinemia in pediatric populations as serum IgG levels below the age-specific mean by at least two standard deviations. Levels below 4 g/L are used in to flag potential need for immunoglobulin replacement therapy. Adverse effects and complications were recorded, including injection reactions, infections, allergic reactions, etc (According to Supplementary Table S1). The changes of steroids dosage over time in the telitacicept group and the control group were also tracked to assess the steroid-sparing effect of telitacicept.

3. Outcome measures: The primary efficacy indicator was the change of proteinuria from baseline to follow-up, assessed by UPCR or 24-hUP Secondary outcomes included changes in serum albumin and eGFR over time, resolution of hematuria, and improvement of other symptoms. We also evaluated the steroid-sparing effect of telitacicept by tracking the dosage of steroids over time in the acute IgAVN group and the control group. According to IPNA pediatric IgAN /IgAVN guideline [8]. Resolution of proteinuria (UPCR < 200 mg/g or 20 mg/mmol or proteinuria < 100 mg/m^2^ per day or < 0.2 g/day in 24-h collection) based on at least two urine samples collected at least 1 month apart in the presence of normal (≥ 90 mL/min/1.73 m2) or stable eGFR. Complete remission includes, in addition to these features, the resolution of hematuria, defined as a negative dipstick for blood and/or < 5 RBC/high-power microscopic field.

### Statistical analysis

Descriptive statistics were expressed as the means and standard deviations (SDs) for normally distributed data or medians and interquartile ranges (IQRs) for non-normally distributed data. Categorical variables were expressed as frequencies (%). For exploratory pairwise comparisons between baseline and individual follow-up timepoints, paired t-tests were used for normally distributed variables, and Wilcoxon signed-rank tests for non-normally distributed variables. To account for repeated measurements across multiple follow-up visits, longitudinal analyses were performed using linear mixed-effects models (LMMs) with random intercepts for subjects. Time was included as a fixed effect, and post-hoc contrasts were applied to compare changes from baseline at each timepoint. This approach accounts for within-subject correlation and accommodates missing data. Exact p-values, effect sizes (mean or median changes), and 95% confidence intervals (CIs) are reported. All analyses were performed using SPSS 26.0 (IBM Corp.) and R 4.4.3 (R Foundation for Statistical Computing). A two-tailed *p* < 0.05 was considered statistically significant.

## Results

### Population characteristics

A total of twenty four children with IgAV and IgAVN treated with telitacicept were enrolled in this study. The cohort consisted of thirteen boys and eleven girls, including five children with IgAV (at risk of nephritis) and nineteen children with biopsy-confirmed IgAVN (ten acute and nine chronic refractory cases). The baseline characteristics of the patients are detailed in Table 1. All the nineteen children with IgAVN had kidney pathology classification ranging from grade ISKDC I to ISKDC Vb, mostly were grade ISKDC III. In the acute IgAVN group, eight out of ten children were ISKDC IIIa or higher classification. In the chronic IgAVN group, all children were ISKDC IIIa or higher, and with crescent formation in 8 out of 9 children (See Supplementary Table S2).

**Table 1:** Participants demographics and baseline characteristics of participants

Characteristics	IgAV (*n* = 5)	Acute phase IgAVN (*n* = 10)	Chronic phase IgAVN (*n* = 9)
Age, mean ± SD, yrs	9.4 ± 2.97	8.84 ± 3.72	11.11 ± 3.18
Male, n (%)	1 (20%)	9 (90%)	3 (33.3%)
Palpable purpura, n (%)	5 (100%)	10 (100%)	9 (100%)
Joint pain/Arthritis, n (%)	1 (20%)	5 (50%)	1 (11.1%)
GI bleed/distress, n (%)	3 (60%)	7 (70%)	4 (44.4%)
Time since diagnosis before telitacicept treatment, mean ± SD, mo	1.1 ± 0.56	1.57 ± 1.24	35.94 ± 31.62
Follow-up time after telitacicept treatment, mean ± SD, wk	39 ± 13.42	25.8 ± 12.07	37.44 ± 10.91
Previous use of systemic immunosuppressive therapy, n (%)			
P	5 (100%)	8 (80%)	9 (100%)
MP	1 (20%)	3 (30%)	4 (44.4%)
CTX pulses	0	3 (30%)	3 (33.3%)
MMF	0	2 (20%)	7 (77.8%)
Tac	0	1 (10%)	3 (33.3%)
CsA	0	0	1 (11.1%)
Concomitant medications with telitacicept, n (%)			
ACEI/ARB	3 (60%)	5 (50%)	6 (66.7%)
P	5 (100%)	9 (90%)	5 (55.6%)
MMF	0	4 (40%)	2 (22.2%)
Tac	0	1 (10%)	3 (33.3%)
CsA	0	0	1 (11.1%)
Baseline 24-h UP, median (IQR), mg/24 h	/	564.1 (147.7,1518.0)	459.8 (303.3,778.1)
Baseline UPCR, median (IQR), mg/g	/	1149.3 (248.3,2405.5)	322.1 (105.2,857.9)
Baseline serum albumin, mean ± SD, g/L	40.54 ± 4.51	35.99 ± 4.56	42.91 ± 3.96
Baseline eGFR, mean ± SD, ml/min/1.73 m^2^	/	121.97 ± 32.62	113.46 ± 19.44
IgA, mean ± SD, g/L	2.95 ± 1.28	2.53 ± 1.22	2.62 ± 0.76
IgG, mean ± SD, g/L	11.28 ± 5.24	8.31 ± 3.78	8.21 ± 1.84
IgM, mean ± SD, g/L	1.32 ± 0.23	1 ± 0.51	1.12 ± 0.41

ACEI angiotensin-converting enzyme inhibitor, ARB angiotensin 2 receptor blocker, CsA Cyclosporine A, CTX Cyclophosphamide, eGFR estimated glomerular filtration rate, IQR interquartile range, MMF Mycophenolate mofetil, MP Methylprednisolone pulses, P Oral glucocorticoids, Tac Tacrolimus, UP urinary protein, UPCR urinary protein-to-creatinine ratio

### Efficacy

**Fig. 1:**
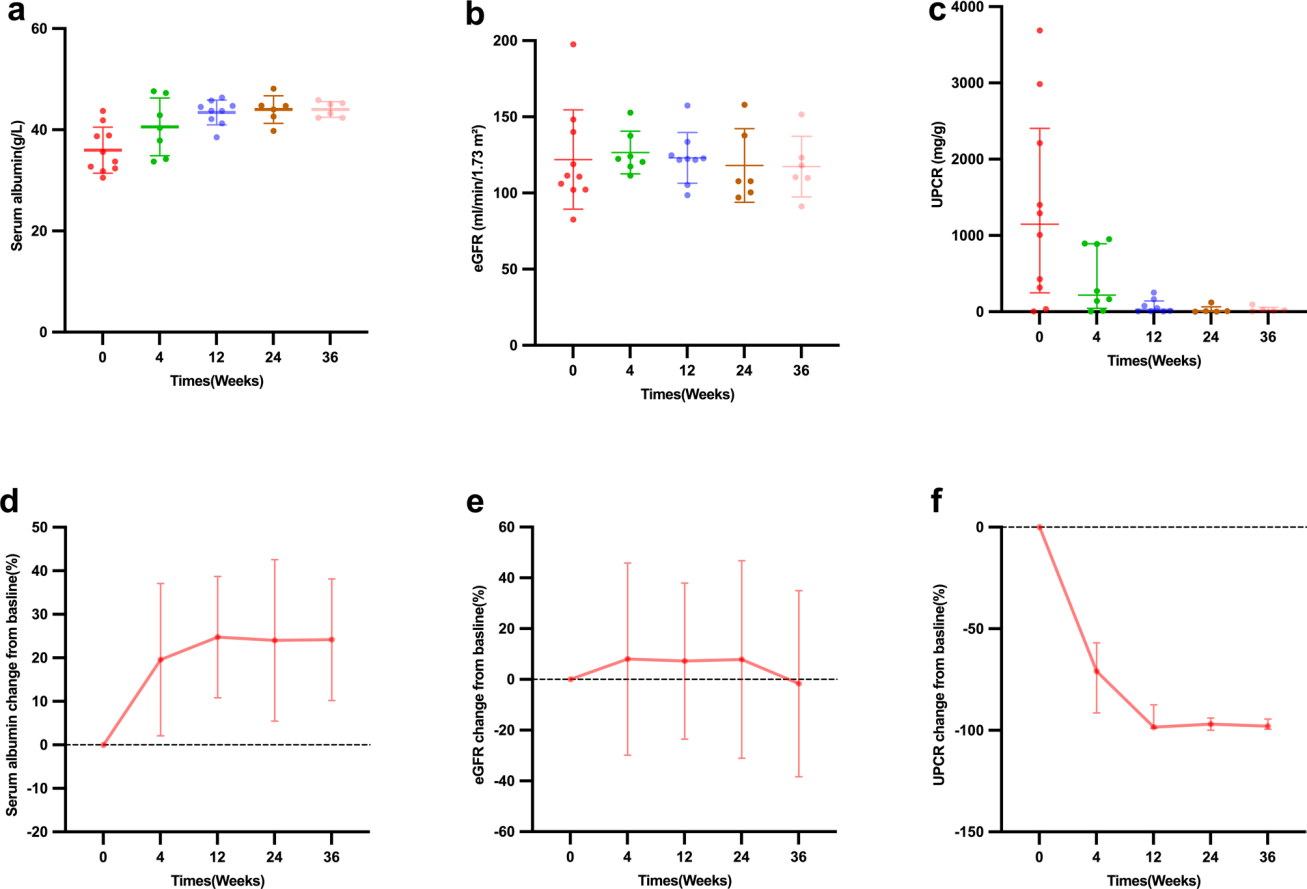
Changes of (**a**, **d**) serum albumin, (**b**, **e**) eGFR and (**c**, **f**) UPCR during treatment with telitacicept in children with acute IgAVN. The error bars indicate (**a**, **b**, **d**, **e**) SD or (**c**, **f**) interquartile range. eGFR estimated glomerular filtration rate, UPCR urine protein-to-creatinine ratio

1. Efficacy in children with IgAV: At baseline, all the children with IgAV had persistent purpura, with three children experiencing severe abdominal pain accompanied by gastrointestinal hemorrhage (melena or hematochezia), one child with arthralgia, and three children with transient hematuria or proteinuria at onset, they were thought to be high-risk for nephritis. They had previously received steroids, and one child received a short course of methylprednisolone pulse(MP) therapy for gastrointestinal hemorrhage, but symptoms were not completely controlled or recurred upon steroids decreased. None of the children with IgAV had received any cytotoxic or biologic therapy before telitacicept. During the mean follow-up period of 39 ± 13.42 weeks, all children with IgAV experienced improvement in skin, joint, and gastrointestinal symptoms, with no kidney involvement observed (Table 2).

2. Efficacy in children with acute IgAVN: Ten children with acute IgAVN received telitacicept in combination with conventional therapy. In the acute IgAVN group, joint and gastrointestinal symptoms significantly improved after telitacicept treatment. The follow-up of the acute IgAVN group is shown in Fig. 1. At baseline, the median UPCR was 1149.3(IQR 248.3–2405.5)mg/g, the mean eGFR was 121.97 ± 32.62 ml/min/1.73 m^2^, the mean albumin was 35.99 ± 4.56 g/L. UPCR significantly decreased at week 4 (*p* = 0.015, Wilcoxon tests) and remained reduced through week 36, eGFR remained stable over time (overall *p* > 0. 05, paired t-tests), with no clinically significant decline observed. Albumin significantly increased at week 4 (*p* = 0.03, paired t-tests) and remained increased through week 36.

**Table 2:** Follow-up of children with IgAV after the use of telitacicept

ID	Gender	Drug dosage (mg)	Follow-up time after telitacicept treatment, wk	Indication for telitacicept use	Follow-up urine test	Side effect
T01	F	160	57	Severe purpura, GI symptoms	Negative	No
T02	F	80	46	Severe purpura, Joint pain	Negative	No
T03	M	80	40	Severe purpura, Transient proteinuria and hematuria	Negative	No
T04	F	160	28	Severe purpura, Transient proteinuria, Severe GI symptoms	Negative	No
T05	F	80	24	Severe purpura, Transient proteinuria and hematuria, Severe GI symptoms	Negative	No

GI gastrointestinal

In longitudinal analysis using LMMs, time was significantly associated with UPCR reduction (overall *p* ≤ 0.02). Compared with baseline, UPCR decreased by − 954 mg/g at week 4 (95% CI − 1603 to − 305, *p* = 0.02), − 1324 at week 12 (95% CI − 2005 to − 643, *p* = 0.001), − 1353 at week 24 (95% CI − 2151 to − 555, *p* = 0.004), and − 1351 at week 36 (95% CI − 2149 to − 553, *p* = 0.005), eGFR remained stable over time (overall *p* > 0. 05, LMM), with no clinically significant decline observed. In longitudinal analysis using a LMM, time was significantly associated with albumin increased (overall *p* ≤ 0.009). Compared with baseline, albumin increased by + 5.15 g/L at week 4 (95% CI 1.98 to 8.32, *p* = 0.009), + 7.75 at week 12 (95% CI 4.82 to 10.69, *p* < 0.001), + 8.09 at week 24 (95% CI 4.69 to 11.49, *p* < 0.001), and + 8.11 at week 36 (95% CI 4.71 to 11.51, *p* < 0.001), eGFR remained stable over time (overall *p* > 0. 05, LMM), with no clinically significant decline observed.

**Fig. 2:**
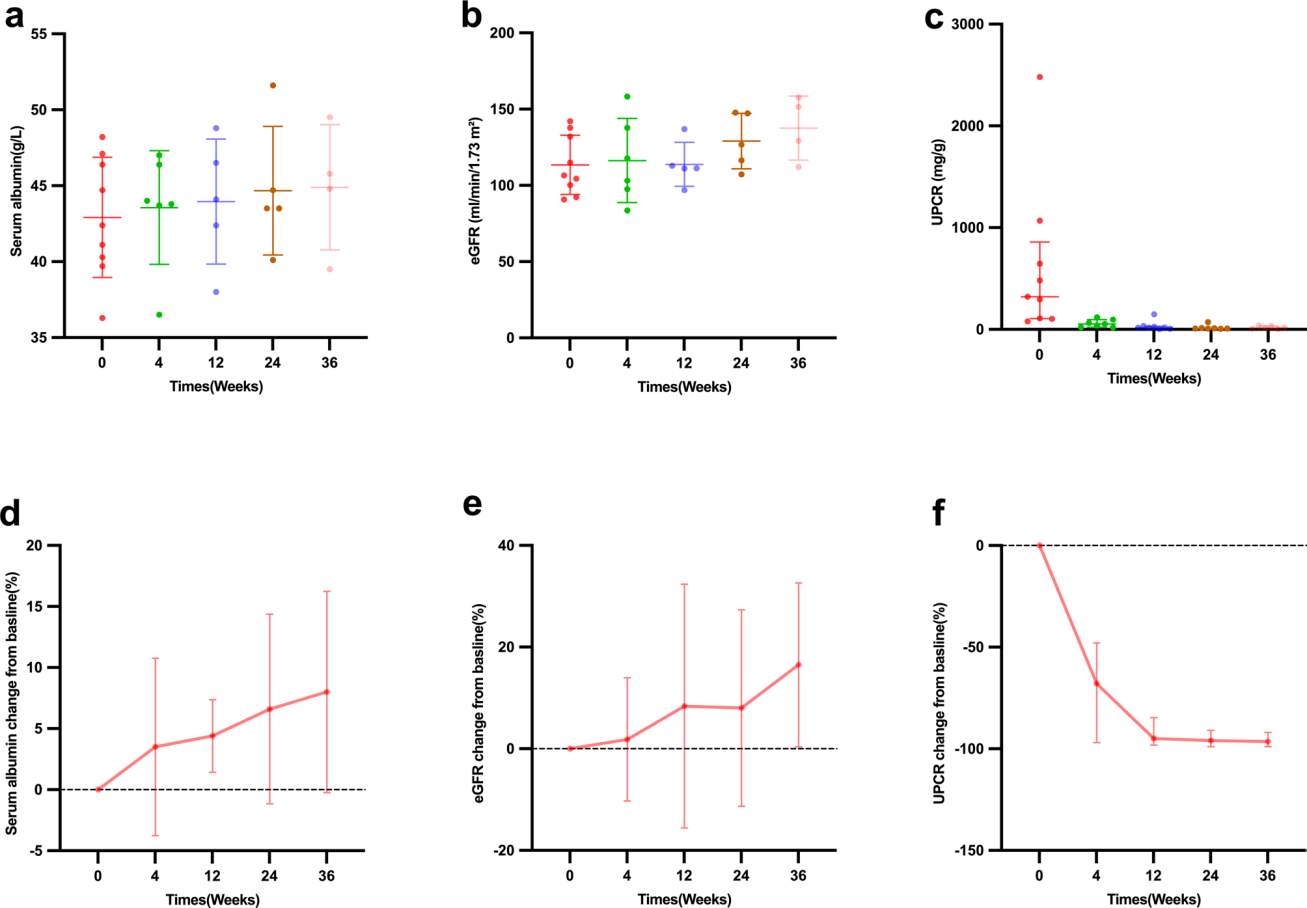
Changes of (**a**, **d**) serum albumin, (**b**, **e**) eGFR and (**c**, **f**) UPCR during treatment with telitacicept in children with chronic IgAVN. The error bars indicate (**a**, **b**, **d**, **e**) SD or (**c**, **f**) interquartile range. eGFR estimated glomerular filtration rate, UPCR urine protein-to-creatinine ratio

3. Efficacy in children with chronic IgAVN: Nine children with chronic refractory IgAVN received telitacicept. The follow-up of the acute IgAVN group is shown in Fig. 2. At baseline, the median UPCR was 322.1(IQR 105.2–857.9)mg/g, the mean eGFR was 113.46 ± 19.44 ml/min/1.73 m2, the mean albumin was 42.91 ± 3.96 g/L. UPCR significantly decreased at week 4 (*p* = 0.028, Wilcoxon tests) and remained reduced through week 36, eGFR remained stable over time (overall *p* > 0. 05, paired t-tests), with no clinically significant decline observed, albumin significantly increased at week 12 (*p* = 0.026, paired t-tests).

In longitudinal analysis using LMMs, compared with baseline, UPCR decreased by − 562 mg/g at week 4 (95% CI − 951 to − 173, *p* = 0.028), − 587 at week 12 (95% CI − 959 to − 215, *p* = 0.016), − 602 at week 24 (95% CI − 992 to − 212, *p* = 0.017), and − 602 at week 36 (95% CI − 1012 to − 192, *p* = 0.024), eGFR remained stable over time (overall *p* > 0. 05, LMM). In longitudinal analysis using a LMM, compared with baseline, albumin increased by + 3.68 g/L at week 36 (95% CI 1.25 to 6.11, *p* = 0.03).

**Fig. 3:**
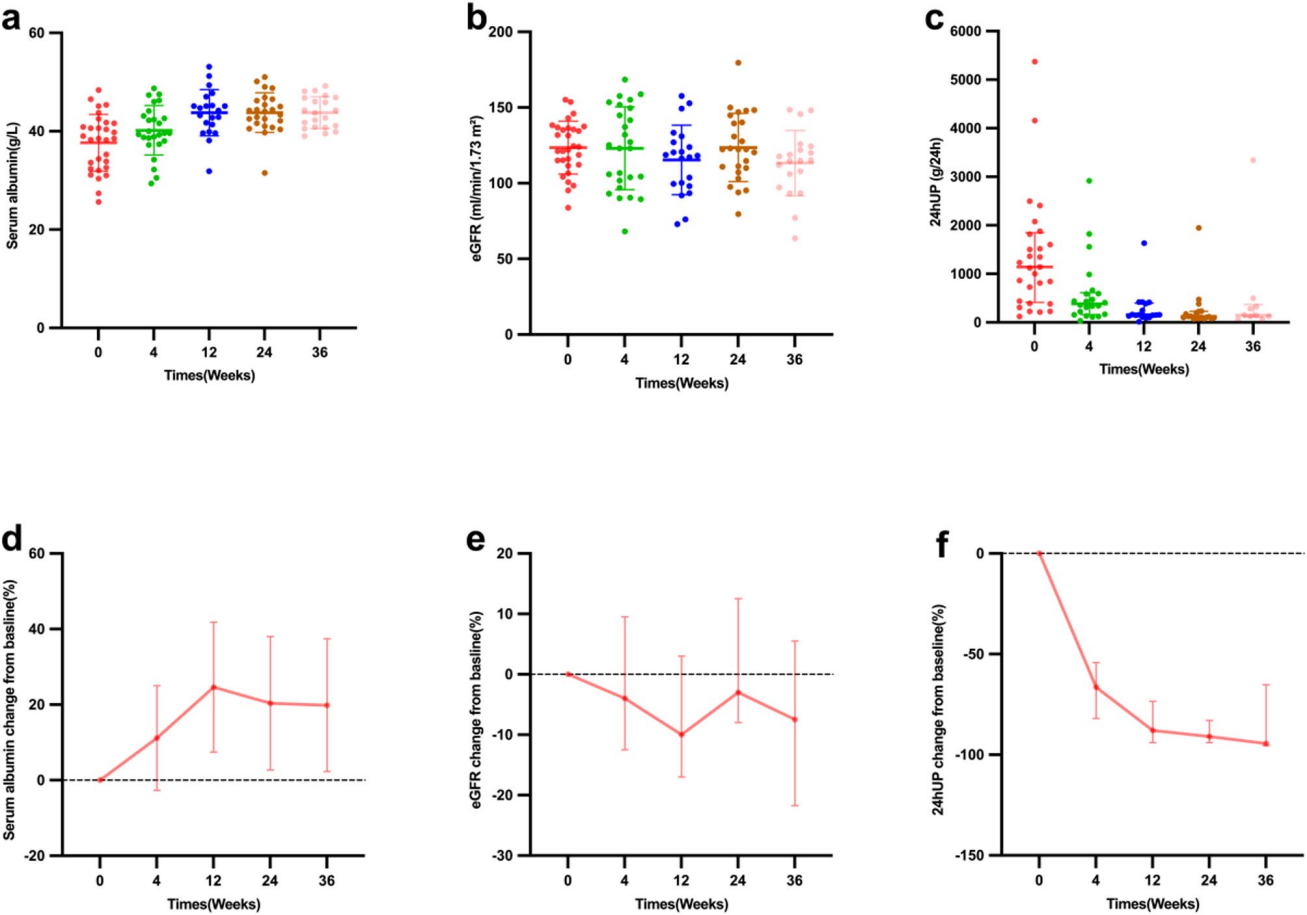
Changes of (**a**, **d**) serum albumin, (**b**, **e**) eGFR and (**c**, **f**) 24hUP in control group. The error bars indicate (**a**, **d**) SD or (**b**, **c**, **e**, **f**) interquartile range. eGFR estimated glomerular filtration rate, UP urinary protein

4. Control group: After matching by PSM, standardized mean differences (SMDs) were < 0.1 for all variables, indicating good baseline balance (see Supplementary Tables S3 and S4). Thirty patients were enrolled in the control group. The follow-up of the control group is shown in Fig. 3. At baseline, the median 24-hUP was 1141.9(IQR 414.5–1845.7)mg/24 h, the mean eGFR was 123.55 ± 17.4 ml/min/1.73 m2, the mean albumin was 37.65 ± 5.78 g/L. 24-hUP significantly decreased at week 4 (*p* < 0.001, Wilcoxon tests) and remained reduced through week 36, eGFR remained stable over time (overall *p* > 0. 05, paired t-tests), albumin significantly increased at week 4 (*p* < 0.001, paired t-tests) and remained increased through week 36.

In longitudinal analysis using LMM, time was significantly associated with 24-hUP reduction (overall *p* < 0.001). Compared with baseline, 24-hUP decreased by − 959 mg/g at week 4 (95% CI − 1442 to − 476, *p* < 0.001), − 1335 at week 12 (95% CI − 1865 to − 805, *p* < 0.001), − 1341 at week 24 (95% CI − 1851 to − 831, *p* < 0.001), and − 1322 at week 36 (95% CI − 1970 to − 674, *p* < 0.001), eGFR remained stable over time (overall *p* > 0. 05, LMM). In longitudinal analysis using a LMM, time was significantly associated with albumin increased (overall *p* ≤ 0.004). Compared with baseline, albumin increased by + 3.00 g/L at week 4 (95% CI 1.21 to 4.79, *p* = 0.004), + 7.00 at week 12 (95% CI 5.08 to 8.92, *p* < 0.001), + 6.58 at week 24 (95% CI 4.77 to 8.39, *p* < 0.001), and + 6.42 at week 36 (95% CI 4.49 to 8.35, *p* < 0.001).

**Fig. 4:**
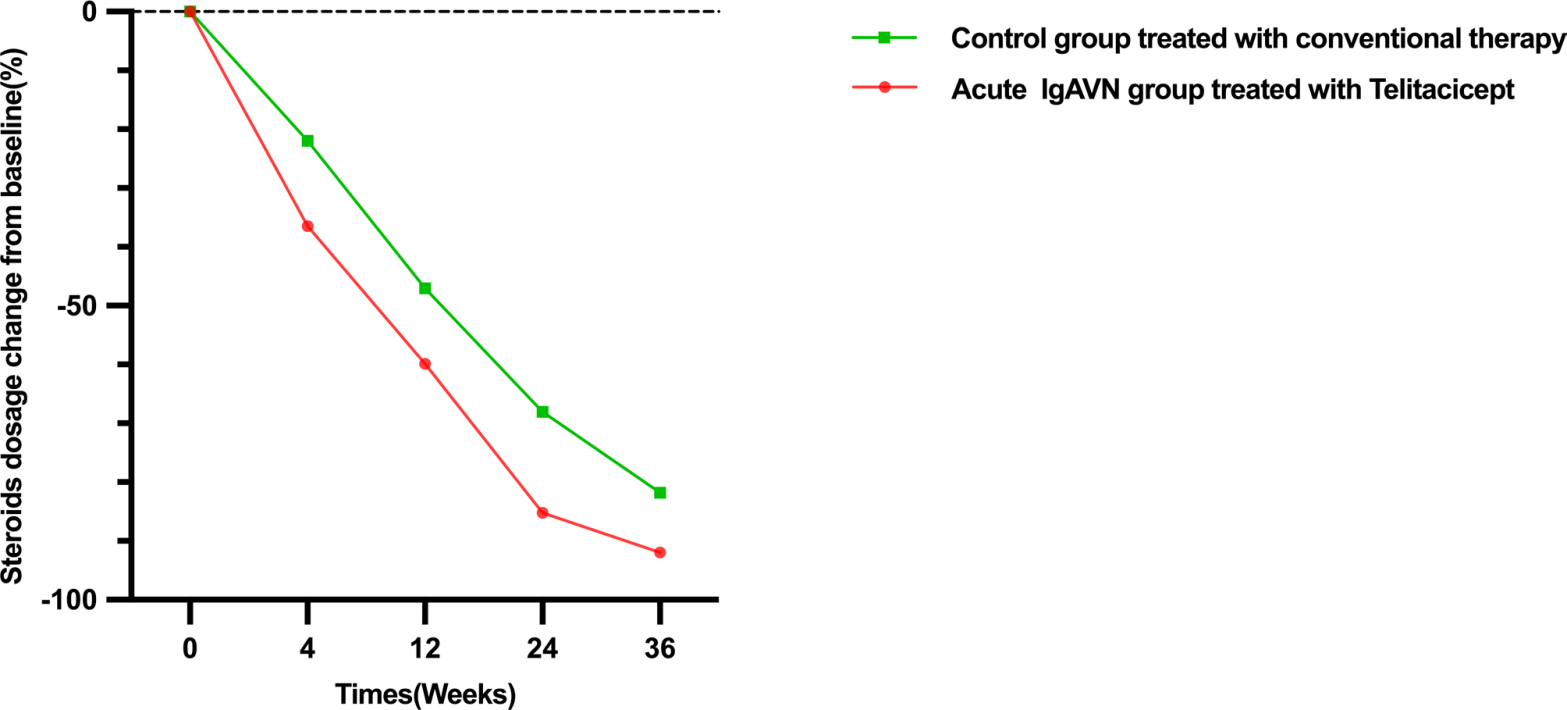
Steroids dosage change of acute IgAVN group (telitacicept treated) and control group (conventional therapy)

By using a repeated-measures LMM (random slope: patient ID; Kenward-Roger degrees of freedom; Bonferroni correction), a significant interaction between acute IgAVN group and time on percentage steroid reduction (F = 4.23, *P* = 0.003) was found. Specifically, the telitacicept group exhibited significantly greater steroid reduction between weeks 4 and 24 compared with controls, suggesting that telitacicept may facilitate more rapid steroid tapering when added to conventional therapy(see Fig. 4; Table 3).

Supplementary Table S5 lists the UPCR trajectories and medication history of the acute IgAVN group, while Supplementary Table S6 lists the 24-hUP levels and medication history of the control group. There were differences in the combination and intensity of therapy. In acute IgAVN group, telitacicept was combined with MMF in four children (4/10) and with Tacrolimus (Tac) in one case (1/10). In comparison, control group had a higher frequency of MMF use (22/30), with six children using Tac (7/30) and three child using cyclosporine(CsA) (3/30).

### Safety

**Fig. 5:**
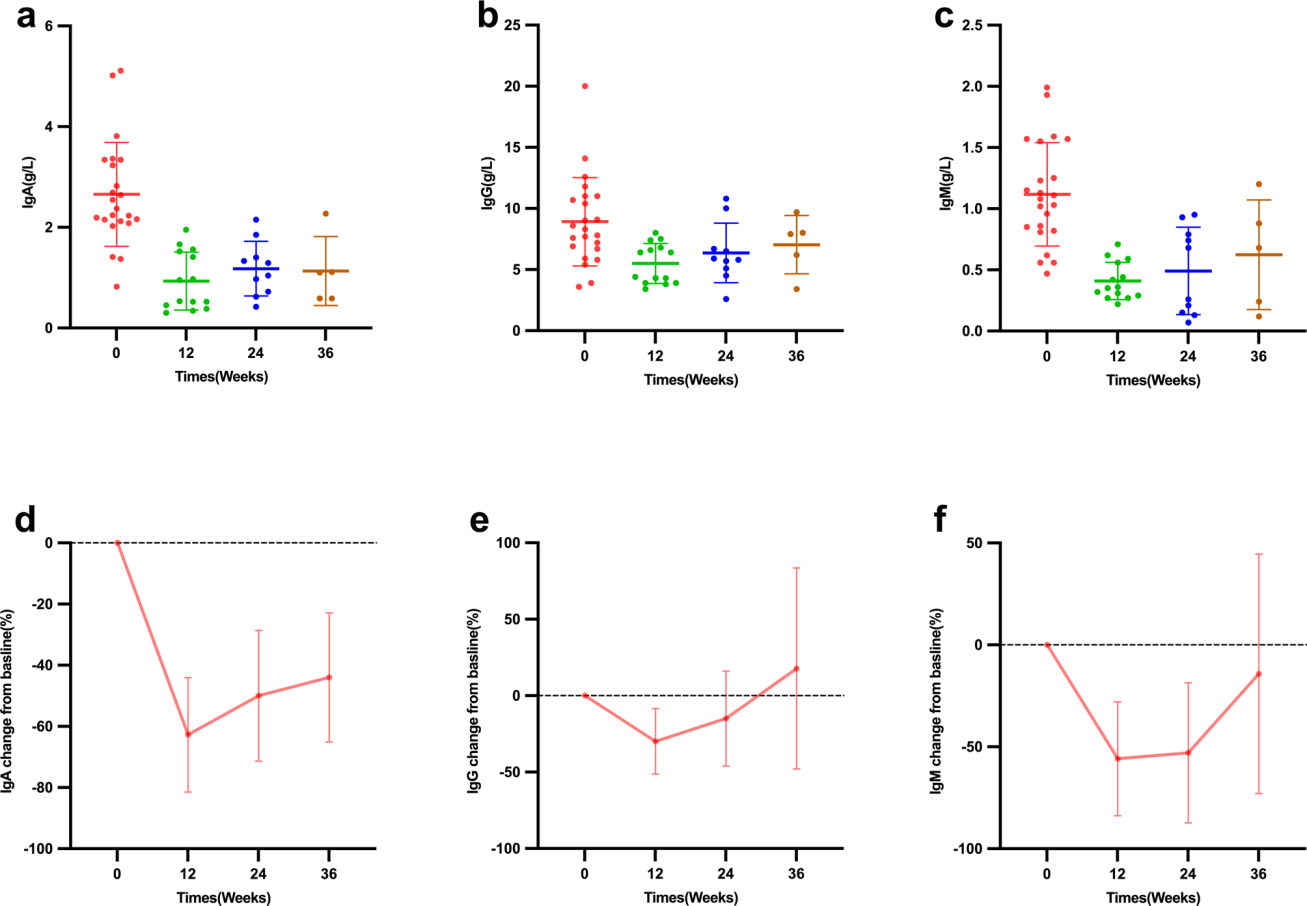
Changes of (**a**) IgA, (**b**) IgG and (**c**) IgM during treatment with telitacicept in children with IgAV and IgAVN. The error bars indicate (**d**, **e**, **f**) SD

During the study, no serious adverse events were observed in the twenty four children. Specifically, injection site reactions, acute allergic reactions and severe infections were not reported. The follow-up of serum immunoglobulin levels is shown in Fig. 5. The LMM was used to assess the changes in IgA, IgG, and IgM at 12, 24, and 36 weeks compared to baseline. The results showed that IgA significantly decreased at week 12 (–1.6870, 95% CI − 2.7273 to − 0.6467, *P* = 0.0052), but the decreases at weeks 24 and 36 were not significant. IgG significantly decreased at weeks 12 and 24 (–3.3745, 95% CI − 4.4148 to − 2.3342, *P* < 0.0001 and − 2.2515, 95% CI − 3.4263 to − 1.0767, *P* = 0.0007, respectively), but the decrease at week 36 was not significant. Changes in IgM were not significant at any time point (*P* > 0.05). These findings indicate that IgA and IgG showed significant declines at earlier time points, while changes in IgM were not significant. Despite the decrease in immunoglobulin levels, no increase in infection rates was observed during follow-up.

**Table 3:** Steroids sparing of the acute IgAVN group and the control group

Time	Ctrl	Acute IgAVN	Acute IgAVN − Ctrl	95% CI	Corrected *P* value
4 W	22.0%	36.4%	14.4%	(–22.8, − 6.0)	0.001
12 W	47.0%	61.3%	14.4%	(− 24.3, − 4.5)	0.004
24 W	67.9%	88.3%	20.4%	(− 32.5, − 8.3)	0.001
36 W	82.8%	91.6%	8.8%	(− 23.2, 5.6)	0.229

## Discussion

Kidney involvement in IgAV is gradually becoming an important cause of CKD in children. Previous studies have found that abdominal pain, gastrointestinal hemorrhage, arthritis or joint pain, persistent purpura, and purpura recurrence may be risk factors for kidney involvement in children with IgAV [20]. Long-term persistent kidney damage can lead to kidney failure in children, affecting their quality of life and imposing a significant burden on families and society. Therefore, the prevention and treatment of IgAV and IgAVN are crucial. The pathogenesis of IgAVN has not yet been fully elucidated. Research in the pathophysiology of IgAVN has revealed many potential therapeutic targets, including mucosal immunity, B-cell and T-cell regulation, RAAS inhibition, and complement pathway modulation [25]. Given the similarities in the pathogenesis of IgAVN and IgAN, many clinical trials for IgAN may help develop new therapeutic strategies for IgAVN. A Phase II clinical trial of telitacicept in IgAN demonstrated its efficacy in reducing proteinuria and maintaining stable eGFR, thereby improving long-term kidney prognosis [14]. This study focuses on evaluating the efficacy and safety of telitacicept in children with IgAV at risk of renal involvement, acute IgAVN with poor prognostic risk factors, and chronic refractory IgAVN who have not responded to conventional therapy.

In this study, after telitacicept treatment, extrarenal symptoms of five children with IgAV improved during follow-up, and no kidney involvement occurred. It indicates that telitacicept may help prevent IgAV with a high risk of renal involvement from progressing to IgAVN. To date, no studies have explored the use of telitacicept for preventing IgAVN, and our data are preliminary. The observation regarding telitacicept potentially preventing renal involvement in high-risk IgAV is based on only five patients without a control group, and therefore this finding is a hypothesis-generating observation. Certainly, not all children with IgAV will develop into IgAVN even without such treatment, so a potential preventive effect requires validation in larger, controlled studies. Given the benign course observed in IgAV, this may be a direction worth exploring in future research. Fenoglio et al. [26] found that rituximab can effectively improve the prognosis of refractory IgAVN which do not respond to conventional treatment. In this study, one child still had persistent proteinuria after treatment with MP, CTX, and rituximab, indicated that not all patients respond to rituximab. Notably, the acute IgAVN patients in our cohort often presented with severe extrarenal symptoms (severe purpura, arthritis/arthralgia, gastrointestinal involvement) that were not substantially relieved by conventional therapy. Following addition of telitacicept, we observed a marked improvement in these extrarenal manifestations. Morever, A major advantage of telitacicept is its ability to reduce steroids usage. In pediatrics, minimizing steroids exposure is crucial due to long-term side effects. Most children treated with telitacicept discontinued steroids within several months without disease relapse, which is in stark contrast to many cases that had previously used low-dose steroids for a long time or resumed use due to disease recurrence. Liu and Jin [16, 17] also found that telitacicept can effectively reduce proteinuria in children with refractory IgAVN and significantly improve prognosis, which is in line with our findings, so in children with chronic or refractory IgAVN, telitacicept may represent a potential therapeutic option, particularly in the context of limited efficacy of conventional immunosuppressive therapies. Telitacicept is currently best regarded as an add-on therapy in IgAVN, in line with standard practice where corticosteroids and/or conventional immunosuppressants remain the backbone of treatment.

The adverse effects observed in this study was hypogammaglobulinemia, including IgA, IgG, and IgM. By blocking BAFF/APRIL signalling, telitacicept reduces B-cell activation, plasmablast/plasma cell survival and therefore lowers production of IgG, IgA and IgM, which is an effect that is mechanistically predictable and appears early after treatment [27]. Clinical trials and several cohort studies report rapid declines in immunoglobulin levels, often detectable within 4 weeks and persisting through 12–24 weeks. This timing fits the drug’s direct effect on B-cell/plasma-cell compartments and the half-life of circulating immunoglobulins (weeks to months). Several RCTs and open-label studies documented statistically significant reductions in IgG, IgA and IgM at week 4 and at 12 weeks after starting telitacicept [14, 28]. Liu et al. [16] reported decreases in IgG, IgM and IgA, and noted hypogammaglobulinemia in a subset, most declines were manageable but require attention. Case series and small pediatric studies of telitacicept in IgA vasculitis nephritis also reported rapid clinical benefit (proteinuria reduction) accompanied by declines in serum immunoglobulins by 12 weeks, consistent with adult findings [18]. Further close follow-up is needed to be vigilant about potential long-term adverse effects. Skin injection reactions are the most commonly reported adverse effects in telitacicept treatment [29], but no such adverse reaction was observed in this study. A larger sample size and detailed documentation of adverse events before and after treatment are needed.

There are some limitations. It is a single-center retrospective study with small sample size. Although the control group was matched on key variables, it was not randomized and is susceptible to confounding and bias. The follow-up duration is relatively limited and longer-term observation is needed to monitor if remission is sustained and to assess long-term safety. Heterogeneity in background immunosuppressive therapy is a major confounder in our study, and that observed clinical improvements cannot be solely attributed to telitacicept, future studies with larger cohorts could consider statistical adjustment, stratification, or randomized controlled designs to more rigorously evaluate the effect of telitacicept. During the matching of the control group, due to the lack of a contemporary control cohort, sensitivity analyses could not be performed. Additionally, the findings in the IgAV group are descriptive, and it is currently unclear whether these children could have avoided nephritis without telitacicept. Controlled trials are needed to verify the preventive use. Given the small sample size and the lack of a control group, telitacicept may represent a potential option for severe/refractory IgAVN, but further studies are needed to confirm efficacy and safety. B-cell phenotyping, serum BAFF/APRIL levels, and Gd-IgA1 would have provided important biological insights into the action of telitacicept. Unfortunately, these biomarkers were not systematically collected in our cohort. The ISKDC grading criteria for renal biopsy in children with IgAVN mainly rely on the involvement of glomerulus in the biopsy tissue. Although ISKDC remains the most widely applied classification in pediatric IgAVN, it does not fully capture the spectrum of histopathological changes, such as the percentage of crescents, chronicity index, and interstitial fibrosis [2, 30, 31]. Given the similarities between IgAVN and IgAN, an increasing number of studies have applied the Oxford Classification (MEST-C score) to IgAVN. It was also found that indicators reflecting chronic renal lesions such as segmental glomerulosclerosis (S) and interstitial fibrosis or tubular atrophy (T) may be risk factors for renal prognosis, and the Oxford classification is more capable of predicting the long-term prognosis of patients with IgAVN than ISKDC [32–34]. However, for the control group, renal biopsy reports were primarily based on the ISKDC classification, and Oxford scores were not routinely recorded. This heterogeneity precluded direct comparison of Oxford classification features between groups. Morever, the safety monitoring in our study was based primarily on telephone and internet follow-up, which may not fully capture the spectrum of infectious events. Despite these limitations, this study demonstrates that telitacicept may be a valuable addition to the treatment of children with IgAVN. It appears to be particularly suitable for patients who do not respond well to steroids and traditional immunosuppressants. The good performance of telitacicept in children with IgAV at high risk of kidney involvement raises the question of whether early intervention can change the disease course in IgAV. Given the subcutaneous administration and weekly dosing frequency of telitacicept, its use is convenient. Compared with daily oral medications, the lower dosing frequency may enhance compliance of patients. The cost and accessibility of telitacicept may be an issue, while its increasing use in SLE treatment may lead to its wider application in IgAVN. In summary, telitacicept showed efficacy and safety in children with IgAV and IgAVN and our results contribute to the evidence that BAFF/APRIL inhibition can induce remission in antibody-mediated kidney diseases. Telitacicept provides a steroid-sparing treatment strategy, which is particularly important in pediatric. These findings need to be further verified in larger-scale multicenter studies and long-term follow-up to fully establish the role of telitacicept in IgAVN management.

## Conclusion

This study demonstrates that telitacicept is a promising therapy for children with IgAV and IgAVN, effectively inducing proteinuria remission, improving systemic symptoms, and reducing the use of steroids, with favorable safety.

## Supplementary Information

Below is the link to the electronic supplementary material.

Supplementary Material 1

## Data Availability

The datasets used and analysed during the current study are available from the corresponding author on reasonable request.

## Abbreviations

IgAV: Immunoglobulin A vasculitis
IgAVN: Immunoglobulin A vasculitis nephritis
CKD: Chronic kidney disease
ESKD: End Stage of Kidney Disease
UPCR: Urinary protein-to-creatinine ratio
eGFR: Estimated glomerular filtration rate
CTX: Cyclophosphamide
MMF: Mycophenolate mofetil
CNIs: Calcineurin inhibitors
Gd-IgA1: Galactose-deficient IgA1
BAFF: B cell activating factor
APRIL: A proliferation-inducing ligand
IgAN: IgA nephropathy
PSM: Propensity score matching
SDs: Standard deviations
IQRs: Interquartile ranges
LMMs: Linear mixed-effects models
ACEI: Angiotensin-converting enzyme inhibitor
ARB: Angiotensin 2 receptor blocker
CsA: Cyclosporine A
MP: Methylprednisolone pulses
P: Oral glucocorticoids
Tac: Tacrolimus
UP: Urinary protein
