# Supplementary material for: Efficacy and safety of telitacicept in children with IgA vasculitis and IgA vasculitis nephritis: a single-center retrospective study
Source: Pediatr Rheumatol Online J. 2025 Oct 21;23:104. doi: 10.1186/s12969-025-01159-3 (PMC12538842; doi:10.1186/s12969-025-01159-3)
Supplement: Supplementary file 1 — Supplementary Material 1 [file 12969_2025_1159_MOESM1_ESM.docx]

**Supplementary Table S1 Safety Profile of Telitacicept**

| **Mild Adverse Events** | Injection site reactions: erythema, pain, swelling, pruritus |
| --- | --- |
|  | Upper respiratory tract infections (URTIs): nasopharyngitis, pharyngitis, mild bronchitis |
|  | Gastrointestinal symptoms: nausea, abdominal discomfort, diarrhea |
|  | General symptoms: fatigue, headache, dizziness |
| **Moderate Adverse Events** | Lower respiratory tract infections or urinary tract infections: usually non-serious, responsive to antibiotics |
|  | Reduction in serum immunoglobulin levels (IgG, IgA, IgM), typically moderate, but persistent decline warrants monitoring |
|  | Mild hematologic changes: leukopenia, neutropenia, or thrombocytopenia (infrequent) |
|  | Skin manifestations: rash, pruritus beyond injection site |
| **Severe Adverse Events** | Serious infections: pneumonia, sepsis, or opportunistic infections |
|  | Hypogammaglobulinemia with recurrent infections: observed in a minority of patients, sometimes requiring IVIG replacement or discontinuation |
|  | Severe hypersensitivity reactions: rare; anaphylaxis not commonly reported but theoretically possible with fusion protein therapy |

**Supplementary Table S2 Time interval between disease onset and the initiation of telitacicept**

| **Group** | **ID** | **ISKDC** | **MEST-C** | **Time interval between disease onset and the initiation of telitacicept, mo** |
| --- | --- | --- | --- | --- |
| IgAV | T01 | / | / | 1.9 |
|  | T02 | / | / | 0.4 |
|  | T03 | / | / | 0.8 |
|  | T04 | / | / | 1.1 |
|  | T05 | / | / | 1.3 |
| Acute IgAVN | T06 | ISKDC IIIa | M0E1S0T0-C0 | 0.5 |
|  | T07 | ISKDC IIIa | M1E1S0T0-C0 | 1.6 |
|  | T08 | ISKDC IIIa | M0E0S1T0-C1 | 0.3 |
|  | T09 | ISKDC IIIa | M0E1S0T0-C1 | 2.4 |
|  | T10 | ISKDC II | M0E1S0T0-C0 | 2.9 |
|  | T11 | ISKDC IVb | M0E1S1T0-C1 | 1.6 |
|  | T12 | ISKDC IIIb | M1E1S1T0-C1 | 1.6 |
|  | T13 | ISKDC Vb | M0E1S0T0-C2 | 4.0 |
|  | T14 | ISKDC IIIa | M1E1S0T0-C0 | 0.3 |
|  | T15 | ISKDC I | M0E0S0T0-C0 | 0.5 |
| Chronic IgAVN | T16 | ISKDC IIIb | M1E0S1T1-C1 | 94.0 |
|  | T17 | ISKDC IIIa | M0E0S0T0-C0 | 72.0 |
|  | T18 | ISKDC IIIa | M0E0S1T0-C1 | 60.0 |
|  | T19 | ISKDC IIIa | M0E0S0T0-C1 | 7.0 |
|  | T20 | ISKDC IIIb | M0E0S1T0-C2 | 8.0 |
|  | T21 | ISKDC IIIa | M0E0S0T0-C1 | 20.0 |
|  | T22 | ISKDC IIIa | M0E0S1T0-C1 | 9.5 |
|  | T23 | ISKDC IVa | M1E0S1T1-C2 | 27.0 |
|  | T24 | ISKDC IIIa | M0E0S0T0-C1 | 26.0 |

**Supplementary Table S3 Demographics and baseline of acute IgAVN group and control group**

| **Characteristics** | **Acute IgAVN Group（n=10）** | **Control Group（n=30）** |
| --- | --- | --- |
| Age, median(IQR), yrs | 8.84 ± 3.72 | 9.21±3.43 |
| Male, n(%) | 9（90%） | 27（90%） |
| Baseline 24-h UP, median (IQR), mg/24h | 564.1（147.7,1518.0） | 1141.9(414.5,1845.7) |
| Baseline UPCR, median (IQR), mg/g | 1149.3（248.3,2405.5） | 1339.8（559.6,3196.9） |
| Baseline serum albumin, mean ± SD, g/l | 35.99 ± 4.56 | 37.65 ± 5.78 |
| Baseline eGFR, mean ± SD, ml/min/1.73 m^2^ | 121.97 ± 32.62 | 123.55 ± 17.4 |

After 1:3 nearest-neighbor propensity score matching (without replacement, caliper = 0.2), the balance between the telitacicept group and historical controls was evaluated using standardized mean differences (SMD), variance ratios (Var. Ratio), and group means.

**Supplementary Table S4 Summary of matching quality**

| **Variable** | **Mean(Treated)** | **Mean(Control)** | **SMD** | **Var.Ratio** |
| --- | --- | --- | --- | --- |
| Distance | - | - | 0.0824 | 1.2803 |
| Age | 8.84 | 9.21 | -0.0986 | 1.1794 |
| Gender | 0.10 | 0.10 | -0.0937 | 0.9910 |
| Pathology | 3.50 | 3.67 | 0.0000 | - |

Interpretation:

All SMDs were <0.1 in absolute value, indicating excellent balance across covariates.

Variance ratios for continuous variables were all close to 1 and within the acceptable range of 0.5–2, suggesting similar distribution shapes between groups.

Post-matching group means were very similar, confirming successful matching.

**Supplementary Table S5 UPCR trajectories and medication history of children in acute IgAVN group**

| **ID** | **Age(yrs)** | **Gender** | **Medication history or medication before telitacicept** | **Dosage of telitacicept(mg)** | **Concomitant immunosuppressive therapy with telitacicept** | **Baseline UPCR (mg/g)** | **Week 4(mg/g)** | **Week 12(mg/g)** | **Week 24(mg/g)** | **Week 36(mg/g)** |
| --- | --- | --- | --- | --- | --- | --- | --- | --- | --- | --- |
| T06 | 12.0 | M | P | 80 | P, MMF | 33.5 | 10.7 | NA | NA | NA |
| T07 | 12.0 | M | CTX, P | 80 | P | 1008.3 | NA | 6.6 | 0 | 5 |
| T08 | 13.0 | M | P | 80 | P, MMF | 319.9 | 142 | 49.6 | 9.3 | 21.4 |
| T09 | 6.0 | M | P | 80 | P | 427.2 | 4.8 | 5.6 | 0 | 7.3 |
| T10 | 3.0 | M | CTX, P | 80 | P | 1290.3 | 894.3 | 164.7 | NA | NA |
| T11 | 7.0 | M | MP, Tac, MMF, P | 80 | Tac, P | 3689.5 | 952.4 | 78 | 7 | 20.8 |
| T12 | 5.8 | M | MP, MMF | 80 | MMF, P | 1402.3 | 270.2 | 13.2 | NA | NA |
| T13 | 11.0 | F | MP, CTX | 160 | P | 2212.7 | 889.3 | 251.6 | 122.8 | 94.7 |
| T14 | 5.6 | M | P | 80 | P, MMF | 2983.9 | 163.9 | 9.4 | NA | NA |
| T15 | 13.0 | M | P | 80 | None | 6.2 | NA | NA | NA | NA |

CsA Cyclosporine A, CTX Cyclophosphamide, MMF Mycophenolate mofetil, MP Methylprednisolone pulses, NA Not Available, P Oral glucocorticoids, Tac Tacrolimus

**Supplementary Table S6 24-hUP levels and medication history of children in control group**

| **ID** | **Age(yrs)** | **Gender** | **Pathology** | **Medication** | **Baseline 24-h UP (g/24h)** | **Week 4 (g/24h)** | **Week 12 (g/24h)** | **Week 24 (g/24h)** | **Week 36 (g/24h)** |
| --- | --- | --- | --- | --- | --- | --- | --- | --- | --- |
| C01 | 10.5 | M | ISKDC IIIa | P, MMF, CsA | 811.9 | NA | NA | 380 | 322 |
| C02 | 5.7 | M | ISKDC IIIa | P, MMF | 1234.3 | 435.6 | NA | 73.7 | 66 |
| C03 | 6.7 | M | ISKDC IIb | MP, CTX, P | 2498.2 | 662.2 | 88 | 100.1 | NA |
| C04 | 7.0 | M | ISKDC IVb | MP, P, Tac | 2079.1 | 470.4 | 417.6 | 101.3 | 123.2 |
| C05 | 5.8 | M | ISKDC IIIb | MP, CTX, P, MMF | 1348.8 | 340 | 132.3 | 75 | 61.2 |
| C06 | 11.8 | F | ISKDC Vb | MP, Tac, P | 5373.7 | 2919.2 | 1633.9 | 472.4 | 499.5 |
| C07 | 12.2 | M | ISKDC IIa | P | 121.8 | NA | NA | NA | NA |
| C08 | 5.7 | M | ISKDC IIIa | MP, CTX, P, MMF | 1361.6 | 433.5 | 162 | 127.4 | NA |
| C09 | 8.0 | M | ISKDC IIa | P, MMF, CsA | 229.5 | 224 | NA | 226.8 | NA |
| C10 | 6.2 | M | ISKDC IIIb | MP, CTX, P, MMF | 1815.6 | 159.2 | 114 | 72 | NA |
| C11 | 13.0 | M | ISKDC IIIa | P, MMF | 2408.3 | 1821 | 149.5 | 121.8 | 123.9 |
| C12 | 16.0 | M | ISKDC IIIa | P, MMF,Tac | 1875.7 | NA | NA | NA | 3343.2 |
| C13 | 10.8 | M | ISKDC IIIa | P, MMF | 392 | 357 | NA | NA | NA |
| C14 | 5.0 | F | ISKDC Va | MP, CTX, P, MMF | 861.8 | 335.3 | 248 | 115.2 | 282.4 |
| C15 | 12.0 | M | ISKDC IIIa | P, MMF | 306.5 | 120 | NA | 100.8 | NA |
| C16 | 5.4 | M | ISKDC IIIa | MP, P, MMF | 841.8 | NA | NA | NA | NA |
| C17 | 11.7 | M | ISKDC IIIa | P, MMF,Tac | 4157 | 592.8 | 385 | 233.8 | 140.4 |
| C18 | 17.0 | F | ISKDC Vb | P, MMF,Tac | 6572.8 | 986 | 414.7 | 1948.2 | 153 |
| C19 | 7.5 | M | ISKDC IVb | MP, P, Tac | 1124.4 | 408 | 155.4 | 78 | NA |
| C20 | 12.4 | M | ISKDC IIIa | P | 437 | 131.3 | 145.3 | NA | NA |
| C21 | 11.5 | M | ISKDC IIIa | P, MMF | 1503.8 | 1559.2 | 150.1 | 121.6 | NA |
| C22 | 6.1 | M | ISKDC IIIb | P, MMF | 1000.1 | 34.2 | 14.5 | 172.4 | NA |
| C23 | 5.8 | M | ISKDC IIIa | P, MMF | 728.5 | NA | 159.6 | 99.9 | NA |
| C24 | 6.4 | M | ISKDC IVb | P, CsA | 1518.4 | 586 | NA | NA | NA |
| C25 | 8.0 | M | ISKDC IIb | P, MMF | 384.8 | 165.6 | 104 | NA | NA |
| C26 | 6.0 | M | ISKDC IIIa | MP, CTX, P, MMF | NA | NA | NA | NA | NA |
| C27 | 6.1 | M | ISKDC IIIa | P | 227.1 | NA | NA | NA | NA |
| C28 | 12.9 | M | ISKDC IIIa | P, MMF,Tac | 1602.7 | 311.9 | 416 | NA | NA |
| C29 | 10.5 | M | ISKDC IIIa | P, MMF | 1141.9 | 114 | NA | 197.4 | NA |
| C30 | 12.5 | M | ISKDC IIIa | P, MMF | 212.8 | NA | NA | NA | NA |

CsA Cyclosporine A, CTX Cyclophosphamide, MMF Mycophenolate mofetil, MP Methylprednisolone pulses, NA Not Available, P Oral glucocorticoids, Tac Tacrolimus
